# Blister fluid as a cellular input for *ex vivo* diagnostics in drug-induced severe cutaneous adverse reactions improves sensitivity and explores immunopathogenesis

**DOI:** 10.1016/j.jacig.2021.11.001

**Published:** 2021-11-30

**Authors:** Andrew Awad, Effie Mouhtouris, Catriona Vi Nguyen-Robertson, Natasha Holmes, Kyra Y.L. Chua, Ana Copaescu, Fiona James, Michelle S. Goh, Ar Kar. Aung, Dale I. Godfrey, Elizabeth J. Philips, Andrew Gibson, Catarina F. Almeida, Jason A. Trubiano, Australian Registry of Severe Cutaneous Adverse Reactions (AUS-SCAR)

**Affiliations:** a Centre for Antibiotic Allergy and Research, Department of Infectious Diseases, Austin Hospital, Heidelberg, Australia; b Department of Microbiology and Immunology, The University of Melbourne, at the Peter Doherty Institute for Infection and Immunity, Melbourne, Australia; c Department of Dermatology, Alfred Health, Melbourne, Australia; d Department of General Medicine, Alfred Health, Melbourne, Australia; e The Institute for Immunology and Infectious Diseases, Murdoch University, Murdoch, Australia; f Department of Medicine, Austin Health, The University of Melbourne, Heidelberg, Australia

**Keywords:** Severe cutaneous drug reactions, *ex vivo* assays, PBMC, BFC

## Abstract

**Background:**

Drug-induced severe cutaneous adverse reactions (SCARs) are presumed T-cell–mediated hypersensitivities associated with significant morbidity and mortality. Traditional *in vivo* testing methods, such as patch or intradermal testing, are limited by a lack of standardization and poor sensitivity. Modern approaches to testing include measurement of IFN-γ release from patient PBMCs stimulated with the suspected causative drug.

**Objective:**

We sought to improve *ex vivo* diagnostics for drug-induced SCARs by comparing enzyme-linked immunospot (ELISpot) sensitivities and flow cytometry–based intracellular cytokine staining and determination of the cellular composition of separate samples (PBMCs or blister fluid cells [BFCs]) from the same donor.

**Methods:**

ELISpot and flow cytometry analyses of IFN-γ release were performed on donor-matched PBMC and BFC samples from 4 patients with SCARs with distinct drug hypersensitivity.

**Results:**

Immune responses to suspected drugs were detected in both the PBMC and BFC samples of 2 donors (donor patient 1 in response to ceftriaxone and case patient 4 in response to oxypurinol), with BFCs eliciting stronger responses. For the other 2 donors, only BFC samples showed a response to meloxicam (case patient 2) or sulfamethoxazole and its 4-nitro metabolite (case patient 3). Consistently, flow cytometry revealed a greater proportion of IFN-γ–secreting cells in the BFCs than in the PBMCs. The BFCs from case patient 3 were also enriched for memory, activation, and/or tissue recruitment markers over the PBMCs.

**Conclusion:**

Analysis of BFC samples for drug hypersensitivity diagnostics offers a higher sensitivity for detecting positive responses than does analysis of PBMC samples. This is consistent with recruitment (and enrichment) of cytokine-secreting cells with a memory/activated phenotype into blisters.

## Introduction

Delayed drug-induced hypersensitivities are a group of presumed conventional T-cell–mediated reactions that range from mild skin conditions (eg, maculopapular exanthema) to severe cutaneous adverse reactions (SCARs) associated with significant morbidity and mortality.^1^ Traditional *in vivo* skin testing techniques such as patch testing or intradermal testing are limited by an absence of standardization, risk of disease relapse, and ill-defined drug testing concentrations.^2^ These limitations can affect the sensitivity of such tests, with published studies suggesting sensitivity ranging from 58% to 64% for acute generalized exanthematous pustulosis, 32% to 80% for drug reactions with eosinophilia and systemic symptoms, and 9% to 24% for Stevens-Johnson syndrome (SJS) and toxic epidermal necrolysis (TEN).^3^ There is also drug-associated variability in patch testing, with β-lactams displaying higher sensitivities and allopurinol and its active metabolite, oxypurinol, exhibiting very low sensitivities.^4^ Evolving approaches include *ex vivo* assays, such as the enzyme-linked immunospot (ELISpot), which detects IFN-γ release following drug challenge. Traditionally, ELISpot assays use the patient’s PBMCs stimulated with the candidate drug to measure cytokine output. The ELISpot assay method is advantageous, as patients are not subjected to additional risk through drug reexposure. Our recent data suggest that IFN-γ release ELISpot assay is an effective diagnostic tool with a 52% to 68% sensitivity and 100% specificity in patients with SCAR.^5^^,^^6^

ELISpot assays detect cytokine (typically IFN-γ) release, which is presumed to be produced by CD4^+^ or CD8^+^ T cells, in patient PBMCs or blister fluid cells (BFCs) following *ex vivo* stimulation with the candidate drug. Cytokine secretion is measured as the number of spot-forming units per million cytokine-secreting cells. Previous case reports have suggested a diminished PBMC IFN-γ ELIspot response over time from SCAR onset, highlighting the importance of performing assays during the acute phase of drug reactions.^7^ This diminished response in peripheral blood may be associated with the lack of a key cell population known as CD8^+^ T tissue-resident memory (TRM) cells, which reside within the dermoepidermal junction, and drug-reactive CD8^+^ T cells are gradually lost from peripheral blood during the recovery period.^8^ In contrast, CD8^+^ TRM cells are more likely to be recruited into BFCs in patients with SCAR. One study compared cytokine production between PBMCs and BFCs, noting that there was a higher expression of perforin and granzyme B in BFCs.^9^ This could be due to localized skin CD8^+^ TRM cells mediating the inflammatory response by recruiting memory CD8^+^ T cells from the circulation, and it suggests that ELISpot assays conducted with PBMCs from patients in the late stages of drug reaction could be less sensitive.^6^^,^^7^^,^^10^ Here, we sought to find ways to improve *ex vivo* assay sensitivity in SCAR diagnostics by examining differences in ELISpot results between 2 different cellular sources: PBMCs and BFCs. This study aimed to provide knowledge that will inform future SCAR testing strategies.

For detailed methods, please see the Methods section in this article's Online Repository at www.jaci-global.org.

## Results and discussion

In this study, we included PBMC and BFC samples (which had been cryogenically stored) from 4 patients (case patients 1-4) with confirmed SCAR, including SJS, TEN, drug reactions with eosinophilia and systemic symptoms, and generalized bullous fixed drug eruption identified from previous prospective studies (see the Methods section in the Online Repository). All patients had a Naranjo score of 4 or higher,^11^ a minimum Toxic Epidermal Necrolysis-Specific Severity of Illness (SCORTEN) score of 2, and a minimum Alden score of 4 for SJS/TEN (Table I).^12^, ^13^, ^14^ Case patients 2 and 4 had 1 implicated drug, whereas case patients 1 and 3 had 3 implicated drugs; all case patients were receiving the implicated drug at time of rash onset (Table II^15^). The latency period for case patients (defined as the time between drug commencement and rash onset) ranged from 0 to 38 days, with a median value of 18.5 days. A latency of 0 days was seen when the rash occurred on day 1 (of the start of administration of the implicated drug). The median delays of PBMC and BFC collection for testing were 15.5 (interquartile range = 35.5) and 17 (interquartile range = 31.5) days, respectively. Case patient 4 had a delayed collection latency of 48 days for PBMCs and 49 days for BFCs. Baseline demographics, clinical features, and biologic sampling details are shown in Tables I and II.

**Table I:** Baseline demographics, biologic sampling, and testing of the cohort

Variable	Case patient 1	Case patient 2	Case patient 3	Case patient 4
Age and sex (y, M/F)	88, M	67, F	38, F	67, M
Ethnicity	White	East Asian	Southeast Asian	Indo-Asian
Prior drug hypersensitivity	Nil	Cefalexin (unknown reaction)	Nil	Nil
Charlson comorbidity index (age-adjusted)	6	2	0	7
Immunosuppression∗	Nil	Nil	Prednisolone, 25 mg daily	Splenectomy
SCAR phenotype†	DRESS	GBFDE	TEN	TEN
Phenotypic score∗	RegiScar: 4	N/A	Alden: 4-5	Alden: 6
HLA results				
HLA-A	01:01:01G 03:01:01G	24:02:01G 24:07:01G	11:01:01G 24:02:01G	33:03:01G 33:03:01G
HLA-B	07:02:01G 18:01:01G	35:05:01G 40:02:01G	40:01:01G 44:03:02G	44:03:02G 58:01:01G
HLA-C	07:01:01G 07:02:01G	03:04:01G 04:01:01G	03:04:01G 07:01:01G	03:02:01G 07:01:01G

*DRESS*, Drug rash with eosinophilia and systemic symptoms; *F*, female; *GBFDE*, generalized bullous fixed drug eruption; *IDT*, intradermal testing; *M*, male; *N/A*, not applicable.

∗ The immunocompromised category includes patients who are known to meet any of the following criteria: transplant recipient, hematologic or oncologic malignancy (in the past 5 years), corticosteroid use in a dose of more than 10 mg prednisolone equivalent per day, connective tissue or autoimmune condition, and AIDS.

† Phenotypic scores are used as per previously published criteria for SJS/TEN (Alden)^13^ and DRESS (RegiSCAR).^20^

**Table II:** Implicated drugs, predictive scores, and latency

Case patient	Drugs implicated	Indication	SCORTEN score∗	Alden score	Naranjo score†	Latency‡ (d)	Receiving at time of rash onset§
1	Benzylpenicillin	Bacteremia	N/A	N/A	4	18	Yes
	Ceftriaxone					33	
	Vancomycin					5	
2	Meloxicam	Joint pain	N/A	N/A	9	0	Yes
3	Trimethoprim/sulfamethoxazole	PJP prophylaxis	2	5	4	38	Yes
	Pantoprazole	Gastric ulcer prophylaxis		4		38	
	Atorvastatin	Nephrotic syndrome		4		38	
4	Allopurinol	Gout	4	6	4	19	Yes
	Ibuprofen			5			

*N/A*, Not applicable; *PJP*, *Pneumocystis jiroveci* pneumonia.

∗ SCORTEN score to predict mortality in patients with SJS/TEN.^12^

† Naranjo adverse reaction score for determining the likelihood of an adverse drug reaction actually being due to the implicated drug.^11^

‡ Latency defined as the time between drug commencement and rash onset (days).

§ Receiving implicated drugs at the onset of rash.

**Fig 1:**
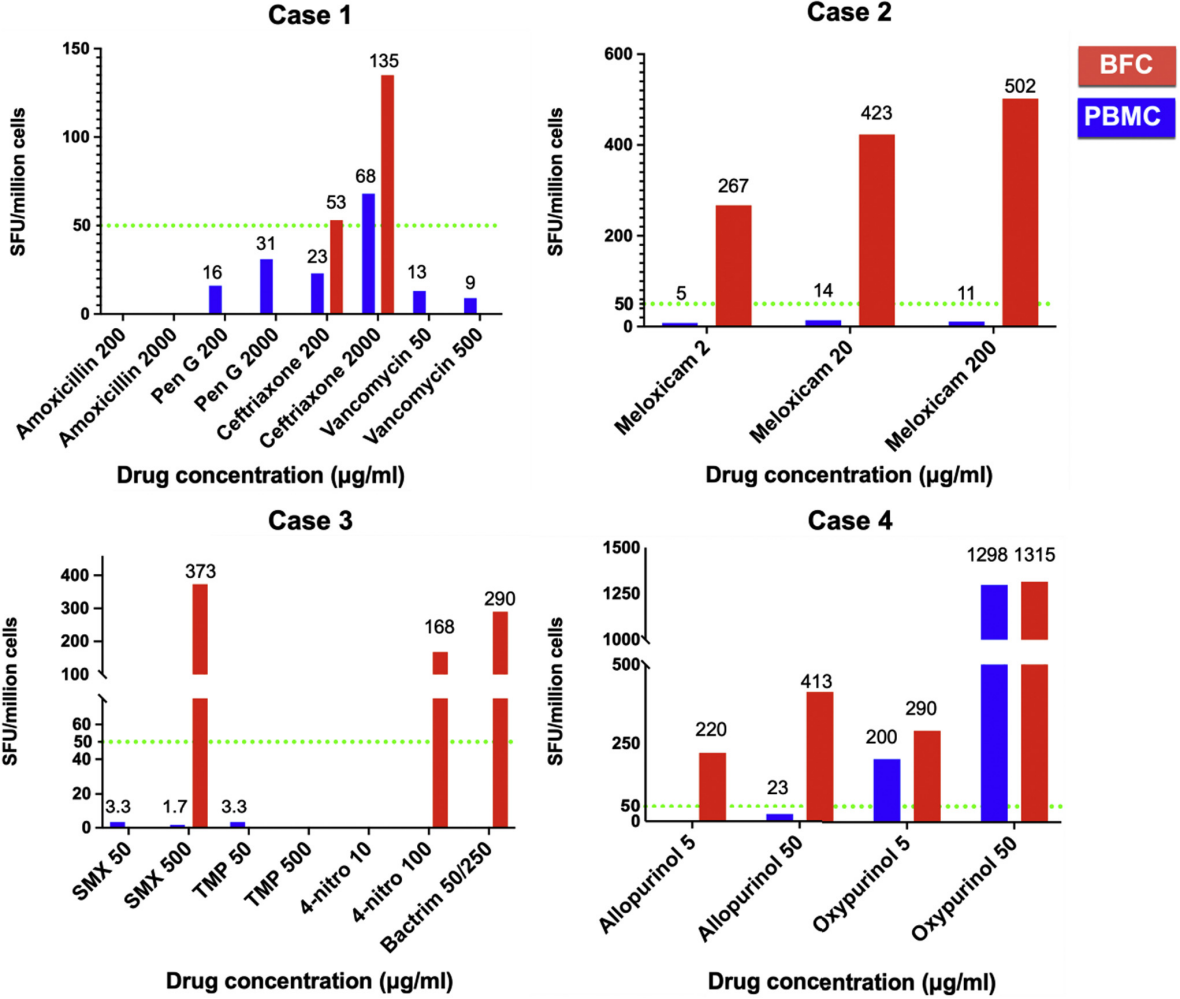
IFN-γ release ELISpot assay release for PBMCs and BFCs. The data are for cryogenically stored PBMC and BFC samples from case patients 1 to 4 (see Table E1 in the Online Repository at www.jaci-global.org). A positive result is defined by at least 50 spot-forming units (SFU) per million cells (*green dotted line*). The maximum doses for each drug were shown to not elicit responses and cell death on a healthy control sample when analyzed by using flow cytometry (7-AAD staining) or lactate dehydrogenase (LDH) viability assay^16^ (see Fig E4 in the Online Repository at www.jaci-global.org). *Pen G*, Penicillin G; *SMX*, sulfamethoxazole; *TMP*, trimethoprim.

IFN-γ ELISpot was performed in matched PBMC and BFC samples from case patients 1 to 4, as per previously published methods^7^ and the procedures described in the Methods section of this article's Online Repository (Figs 1^16^ and 2,^17^^,^^18^ and see Figs E1 and E2 in the Online Repository at www.jaci-global.org). Two of these patients displayed positive ELISpot results (defined as ≥50 spot-forming units per million cells)^7^^,^^8^ after *ex vivo* challenge with suspected drugs for both PBMCs and BFCs (the suspected drug for case patient 1 was ceftriaxone and that for case patient 4 was oxypurinol), whereas case patients 2 and 3 displayed a positive result only with BFCs (Fig 1).

**Fig 2:**
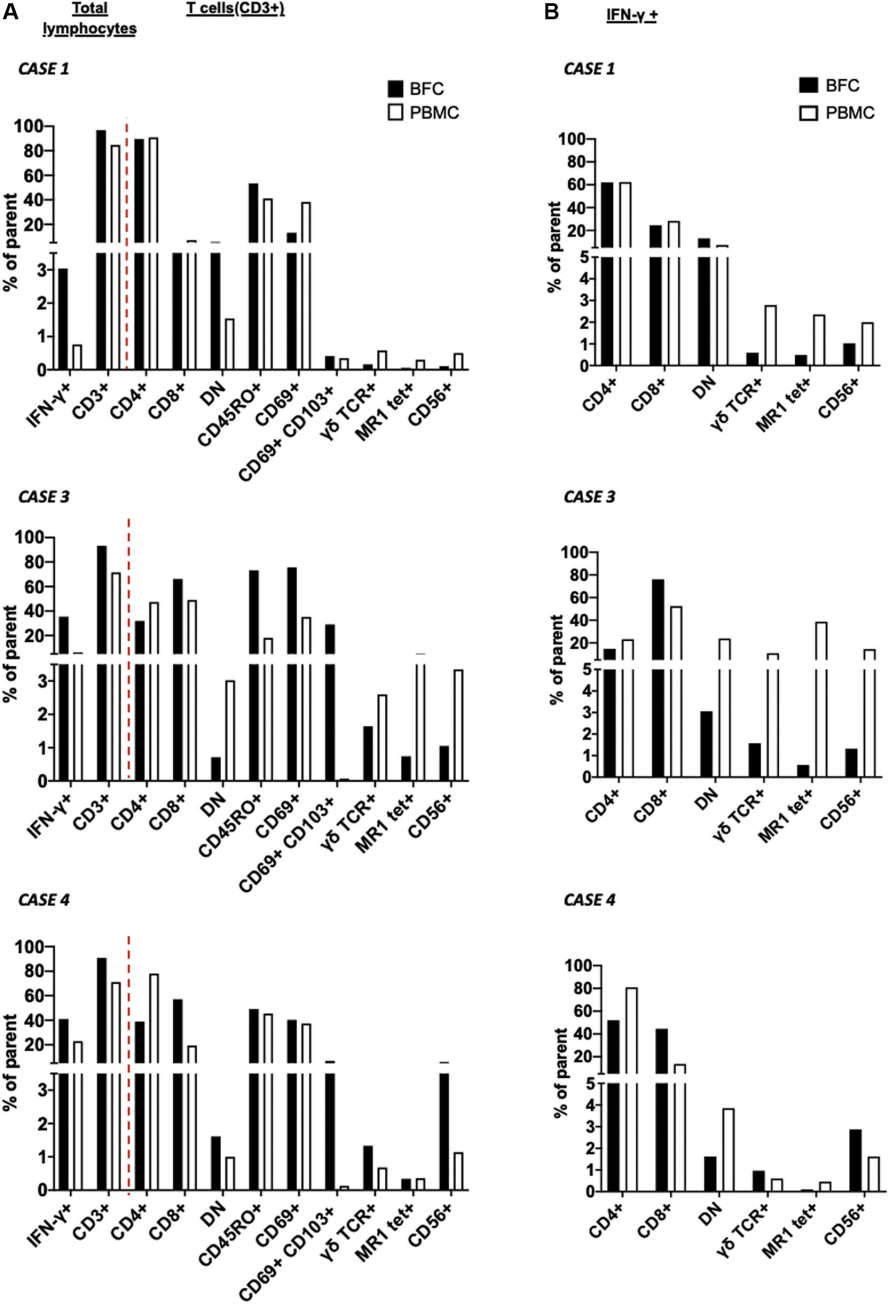
Lymphocyte composition of blood and blister samples. Donor-matched BFCs and PBMCs were analyzed by flow cytometry. **A,** Graphs show the percentages of total IFN-γ–positive and CD3^+^ lymphocytes (*to the left of the red line*) among the total live lymphocytes (gated as per Fig E1, *i*). T cells (CD3^+^) (gated after exclusion of CD14 [monocytes] and CD19 [B cells] as per Fig E1, *ii*) were subsequently analyzed for CD4 and CD8 coreceptors (CD4^–^/CD8^–^ cells are indicated as double-negative [DN]), CD45RO (memory), CD69 (activation), CD69 and CD103 coexpression (egress/tissue residency/memory), γδ T-cell receptor (TCR), binding to MR1 5-OP-RU tetramers^17^^,^^18^ (mucosal-associated invariant T [MAIT] cells), or expression of the NK receptor CD56 (NK-like T cells) (*to the right of the red line*). **B,** Graphs show proportions of CD4, CD8, and CD4/CD8 DN T cells, γδ T cells, MAIT cells, and CD56^+^ T cells among IFN-γ–secreting cells, gated as per Fig E2.

In terms of drug concentration, BFCs or PBMCs were incubated with the candidate drugs at a concentration that represented the peak serum concentration and a level 10- to 20-fold higher than the peak serum concentration. The BFCs of case patient 1 tested positive for both doses of ceftriaxone (200 and 2000 μg/mL), whereas that same patient's matched PBMCs tested positive only to the highest dose, with half of the response elicited in BFCs. The BFCs of case patient 4 tested positive at both concentrations (5 and 50 μg/mL) of allopurinol in addition to its metabolite, oxypurinol, whereas that same patient's PBMCs showed a positive response only to oxypurinol. This suggests that BFC analysis can provide higher sensitivity to drug hypersensitivity testing than PBMC analysis can. This is further supported by analysis of case patients 2 and 3, with positive IFN-γ release ELISpot responses detected with use of BFC samples but not PBMC samples. The BFCs of case patient 2 displayed positivity to all doses of meloxicam (2, 20, and 200 μg/mL), and those of case 3 patient displayed positivity only to the highest dose of sulfamethoxazole (500 μg/mL), its metabolite 4-nitro-sulfamethoxazole (100 μg/mL), and the commercial product trimethoprim-sulfamethoxazole at 50 and 250 μg/mL of the sulfamethoxazole component, respectively.

Flow cytometry was used to investigate whether the different cellular compositions of matched BFCs and PBMCs for case patients 1, 3, and 4 could account for the observed differences in ELISpot sensitivity (Fig 2 and see Figs E1 and E2). We found that the BFC samples were enriched for total T (CD3^+^) cells and for IFN-γ–secreting cells relative to the matched PBMC samples (Fig 2, *A* and see Figs E1 and E2). The total proportions of CD4^+^, CD8^+^, and double-negative T cells varied across individuals (Fig 2, *A* and see Fig E1), likely reflecting differences in the pathology and/or treatments, with case patient 1 displaying a strong bias for CD4^+^ T cells, which is typical of drug reactions with eosinophilia and systemic symptoms.^15^ In contrast, the BFCs of case patient 4 were enriched for CD8^+^ T cells relative to matched PBMCs, which may be associated with a delayed BFC sampling compared with that in the other cases (Table I), possibly reflecting CD8^+^ T cells' egress from the peripheral blood.^8^ The BFCs of case patients 3 and 4 showed an enrichment for T-cell populations with a tissue residency/recruitment (CD69^+^CD103^+^) phenotype, which have been implicated in SCAR^8^ (Fig 2, *A* and see Fig E1). The BFC samples of case patient 3 further displayed higher proportions of memory (CD45RO) and activated (CD69) T cells relative to PBMCs while remaining similar to the samples for case patients 1 and 4, which may partly account for the differences in ELISpot sensitivity between the 2 samples (Fig 1). As unconventional T cells (not HLA-restricted) are also known to produce IFN-γ and their role in SCAR remains unexplored,^14^ we assessed the proportions of γδ T cells, mucosal-associated invariant T cells and CD56-expressing T cells (T cells expressing natural killer [NK] markers, likely including NK T cells).^19^ Although mucosal-associated invariant T cells and γδ T cells did not show preferential recruitment into BFCs (Fig 2, *A* and see Fig E1), they were found among IFN-γ–positive populations (Fig 2, *B* and see Fig E2, *B*), representing large proportions of the PBMCs of case patient 3 (38.4% and 11.4%, respectively). The IFN-γ–secreting cells of the PBMCs of case patient 3 also included NK-like T cells (CD56^+^CD3^+^) and NK cells (CD56^+^CD3^–^). Overall, the IFN-γ–secreting cells comprised CD4^+^, CD8^+^, and double-negative (CD4^–^CD8^–^) T cells, with preferential enrichment for CD8^+^ T cells in BFCs from case patients 3 and 4 and displayed memory and activated phenotypes (CD45RO^+^/CD69^+^) (Fig 2 and see Fig E2, *B*). Overall, the BFC samples displayed T lymphocytes that had been recruited from the blood or adjacent tissue with an activated phenotype and cytokine secretion capacity. This leads to higher proportions of cells with an IFN-γ secretion capacity (than in blood), which may reflect higher representations of the drug antigen-specific clones. Collectively, these results suggest a higher sensitivity for BFC samples in ELISpot testing than for PBMC samples, likely reflecting differences in their cellular composition.

*Ex vivo* drug hypersensitivity diagnostics have an increasing evidence base and clinical demand.^3^ By analyzing samples from 4 patients with SCAR with distinct drug hypersensitivity and clinical manifestations that are presumed to be T-cell–mediated, this study provides impetus for further work to explore alternative sampling sources for drug hypersensitivity diagnostics. At present, there is no criterion standard diagnostic for causality assessment in SCAR, and previous studies, although showing promising sensitivity,^3^ remain limited. Our results suggest higher sensitivity for BFC analysis relative to analysis of matched PBMCs when *ex vivo* IFN-γ release ELISpot is used.^3^ Although our study is limited by low numbers and cell viability, the rare nature of cases of both blister fluid capture and SCAR that have been accurately phenotyped provides a unique insight into the diagnostic potential for this IFN-γ release ELISpot assay.

Our results are consistent with recruitment of known populations involved in the pathology (T cells with a memory/activated phenotype and cytokine-secreting capacity) into blisters. We further reveal that relative to BFCs, PBMCs may have lower representation of cells with an IFN-γ secretion capacity.^7^^,^^8^ How much IFN-γ detection by ELISpot is due to direct activation of drug-specific cells or bystander secretion of nonspecific cells remains to be understood, and it may vary with the drug causing the SCAR. It is possible that following activation, some drug-specific cells may produce cytokines other than IFN-γ (such as TNF, IL-4, and IL-17) that have not been tested. Although we also assessed IL-17 secretion by using flow cytometry, our results do not seem to suggest that this could be a key contributor for the responses studied (see Fig E3 in the Online Repository at www.jaci-global.org). This may require ELISpot assays for other cytokines or markers yet to be identified, or even more generic activation assays using cellular activation markers such as CD69 and CD107a. Thus, we recommend that clinicians sample BFCs, whenever available, for testing with ELISpot assays in drug hypersensitivity diagnostics, while retaining correlation with PBMC results. This may prove to be an invaluable resource for future studies aiming at characterizing the immunopathogenesis and HLA (or HLA-like) restriction of these drug-induced hypersensitivities, including drug presentation pathways, cell populations involved, and cytokine outputs. Such knowledge may ultimately lead to improved diagnostics for patients with SCAR, improving efforts to lower the significant morbidity and mortality associated with SCAR.

**Clinical implications**

Although obtaining blister fluid samples may be less readily available than blood samples, BFC offer higher sensitivity for *ex vivo* drug-hypersensitivity diagnostics compared to PBMC samples.

## Supplementary data

Fig E1

Fig E2

Fig E3

Fig E4

## References

[1] Lin Y.F., Yang C.H., Sindy H., Lin J.Y., Rosaline Hui C.Y., Tsai Y.C. (2014). Severe cutaneous adverse reactions related to systemic antibiotics. Clin Infect Dis.

[2] Phillips E.J., Bigliardi P., Bircher A.J., Broyles A., Chang Y.S., Chung W.H. (2019). Controversies in drug allergy: testing for delayed reactions. J Allergy Clin Immunol.

[3] Copaescu A., Gibson A., Li Y., Trubiano J.A., Phillips E.J. (2021). An updated review of the diagnostic methods in delayed drug hypersensitivity. Front Pharmacol.

[4] Johansen J.D., Aalto-Korte K., Agner T., Andersen K.E., Bircher A., Bruze M. (2015). European Society of Contact Dermatitis guideline for diagnostic patch testing - recommendations on best practice. Contact Dermatitis.

[5] Trubiano J.A., Strautins K., Redwood A.J., Pavlos R., Konvinse K.C., Aung A.K. (2018). The combined utility of ex vivo IFN-γ release enzyme-linked immunospot assay and in vivo skin testing in patients with antibiotic-associated severe cutaneous adverse reactions. J Allergy Clin Immunol Pract.

[6] Copaescu A., Mouhtouris E., Vogrin S., James F., Chua K.Y.L., Holmes N.E. (2021). The role of in vivo and ex vivo diagnostic tools in severe delayed immune-mediated adverse antibiotic drug reactions. J Allergy Clin Immunol Pract.

[7] Trubiano J.A., Redwood A., Strautins K., Pavlos R., Woolnough E., Chang C.C. (2017). Drug-specific upregulation of CD137 on CD8+ T cells aids in the diagnosis of multiple antibiotic toxic epidermal necrolysis. J Allergy Clin Immunol Pract.

[8] Trubiano J.A., Gordon C.L., Castellucci C., Christo S.N., Park S.L., Mouhtouris E. (2020). Analysis of skin-resident memory t cells following drug hypersensitivity reactions. J Invest Dermatol.

[9] Posadas S.J., Padial A., Torres M.J., Mayorga C., Leyva L., Sanchez E. (2002). Delayed reactions to drugs show levels of perforin, granzyme B, and Fas-L to be related to disease severity. J Allergy Clin Immunol.

[10] Tanvarasethee B., Buranapraditkun S., Klaewsongkram J. (2012). The potential of using ELISPOT to diagnose cephalosporin-induced maculopapular exanthems. Acta Derm Venereol.

[11] Naranjo C.A., Busto U., Sellers E.M., Sandor P., Ruiz I., Roberts E.A. (1981). A method for estimating the probability of adverse drug reactions. Clin Pharmacol Ther.

[12] Bastuji-Garin S., Fouchard N., Bertocchi M., Roujeau J.C., Revuz J., Wolkenstein P. (2000). SCORTEN: a severity-of-illness score for toxic epidermal necrolysis. J Invest Dermatol.

[13] Sassolas B., Haddad C., Mockenhaupt M., Dunant A., Liss Y., Bork K. (2010). ALDEN, an algorithm for assessment of drug causality in Stevens-Johnson Syndrome and toxic epidermal necrolysis: comparison with case-control analysis. Clin Pharmacol Ther.

[14] de Lima Moreira M., Souter M.N.T., Chen Z., Loh L., McCluskey J., Pellicci D.G. (2020). Hypersensitivities following allergen antigen recognition by unconventional T cells. Allergy.

[15] Miyagawa F., Asada H. (2021). Current perspective regarding the immunopathogenesis of drug-induced hypersensitivity syndrome/drug reaction with eosinophilia and systemic symptoms (DIHS/DRESS). Int J Mol Sci.

[16] Copaescu A., Choshi P., Pedretti S., Mouhtouris E., Peter J., Trubiano J.A. (2021). Dose dependent antimicrobial cellular cytotoxicity-implications for ex vivo diagnostics. Front Pharmacol.

[17] Reantragoon R., Corbett A.J., Sakala I.G., Gherardin N.A., Furness J.B., Chen Z. (2013). Antigen-loaded MR1 tetramers define T cell receptor heterogeneity in mucosal-associated invariant T cells. J Exp Med.

[18] Corbett A.J., Eckle S.B., Birkinshaw R.W., Liu L., Patel O., Mahony J. (2014). T-cell activation by transitory neo-antigens derived from distinct microbial pathways. Nature.

[19] Godfrey D.I., Uldrich A.P., McCluskey J., Rossjohn J., Moody D.B. (2015). The burgeoning family of unconventional T cells. Nat Immunol.

[20] Kardaun S.H., Sekula P., Valeyrie-Allanore L., Liss Y., Chu C.Y., Creamer D. (2013). Drug reaction with eosinophilia and systemic symptoms (DRESS): an original multisystem adverse drug reaction. Results from the prospective RegiSCAR study. Br J Dermatol.

