# Supplementary material for: Blister fluid as a cellular input for ex vivo diagnostics in drug-induced severe cutaneous adverse reactions improves sensitivity and explores immunopathogenesis
Source: J Allergy Clin Immunol Glob. 2021 Nov 30;1(1):16–21. doi: 10.1016/j.jacig.2021.11.001 (PMC10509900; doi:10.1016/j.jacig.2021.11.001)
Supplement: Fig E4 [file mmc4.docx]

**[drug] = μg/mL**

**[TMP/SMX]**
