# Supplementary material for: Blister fluid as a cellular input for ex vivo diagnostics in drug-induced severe cutaneous adverse reactions improves sensitivity and explores immunopathogenesis
Source: J Allergy Clin Immunol Glob. 2021 Nov 30;1(1):16–21. doi: 10.1016/j.jacig.2021.11.001 (PMC10509900; doi:10.1016/j.jacig.2021.11.001)
Supplement: Supplementary Material [file mmc5.docx]

**Supplementary Materials**

Supplementary Methods 2

*IFN-γ ELISpot Assay* 2

*Flow Cytometry* 2

*Generation of MR1 5-OP-RU tetramers* 2

*Data Collection* 2

References: 3

Supplementary Figure 1. Flow cytometry gating strategy 4

Supplementary Figure 2. Production of IFN-γ by lymphocytes in blood and blister fluid samples 5

Supplementary Figure 3. Production of IL-17 by T cells in blood and blister fluid samples 6

Supplementary Figure 4 Cytotoxicity assays for Sulfamethoxazole (SMX), Trimethoprim (TMP) and Bactrim 7

# **Supplementary Methods**

## ***IFN-γ ELISpot Assay***

Peripheral blood mononuclear cells (PBMC) were isolated from whole heparinised blood and serous fluid was removed from blisters (blister fluid cells, BFC) of SCAR patients recruited in this study. Samples were stored at -80^o^C in 90% heat-inactivated fetal bovine serum (FBS) and 10% dimethyl sulfoxide (DMSO) prior to use. IFN-γ release with co-incubation of the drugs implicated in each patient’s adverse reactions was performed by ELISpot assay in triplicate from thawed or fresh PBMC or BFC.

PBMC or BFC (200,000 cells per well) were incubated with candidate drugs at concentrations that represented peak serum concentration (Cmax) and a level 10 to 20-fold higher than Cmax. The maximum doses of drugs used in this study were shown to not elicit cell death as measured by a Lactate Dehydrogenase (LDH) release colorimetric assay or 7-AAD staining using flow cytometry on a healthy control sample (Supplementary Figure 4 and as per previously published guidelines(1)). Testing was also performed using a negative (unstimulated) and positive control (Anti-CD3 antibody; Mabtech, Victoria, Australia). The mean number of spots for the test and unstimulated wells were calculated. A positive response was defined as greater than or equal to 50 spot-forming unit (SFU)/million cells after background (unstimulated control) removal as per previously published definitions (2, 3).

## ***Flow Cytometry***

Flow cytometry was performed to assess IFN-γ production and surface phenotype of T cells isolated from BFC and PBMC. Cells from Cases 1, 3 and 4 were cultured at 37 °C for 4 hours in supplemented RPMI media alone or with phorbol 12-myristate 13-acetate (PMA) (50 ng/ml) and ionomycin (1 μg/ml), in the presence of Golgi Plug protein transport inhibitor containing Brefeldin A (BD). Cells were subsequently labelled with antibodies or MR1 5OP-RU tetramers diluted in phosphate-buffered saline with 10% FBS (Gibco) for 30 min at room temperature. Antibodies used targeted human γδTCR, CD3, CD4, CD8, CD14, CD19, CD45RO, CD56, CD69, CD103, and 7-aminoactinomycin D (7-AAD) was used as a viability dye. Cells were washed then fixed and permeabilised according to manufacturer’s instructions (BD Biosciences), and then stained with an antibodies targeting IFN-γ and IL-17. Flow cytometry was performed on a BD LSRFortessa and data were analysed using an analysis software (FlowJo LLC).

## ***Generation of MR1 5-OP-RU tetramers***

MR1 5OP-RU tetramers were generated in house as described (4, 5). In brief, truncated ectodomains of human MR1 were expressed as inclusion bodies in *Escherichia coli* (BL21) along with β_2_-microglobulin (β2m). MR1 and β2m inclusion bodies were then refolded in vitro in the presence or absence of 5-A-RU (provided by Gavin Painter, University of Queensland) and methylglyoxal (Sigma), using oxidative refolding, prior to dialysis with subsequent Ni-NTA agarose and anion-exchange chromatography. MR1 monomers underwent chemical biotinylation prior to storage at −80 °C. Monomers were tetramerised using streptavidin-BV421 (BD).

## ***Data Collection***

Cases with cryogenically stored PBMC and BFC were identified from previous prospective ethically approved studies (PIPA: HREC/15/Austin/75; AUS-SCAR: HREC/50791/Austin-2019, Melbourne, Australia.

# **References:**

1. Copaescu A, Choshi P, Pedretti S, Mouhtouris E, Peter J, Trubiano JA. Dose Dependent Antimicrobial Cellular Cytotoxicity—Implications for ex vivo Diagnostics. Frontiers in Pharmacology. 2021;12(2069).

2. Copaescu A, Mouhtouris E, Vogrin S, James F, Chua KYL, Holmes NE, et al. The Role of In Vivo and Ex Vivo Diagnostic Tools in Severe Delayed Immune-Mediated Adverse Antibiotic Drug Reactions. J Allergy Clin Immunol Pract. 2021;9(5):2010-5.e4.

3. Porebski G. In Vitro Assays in Severe Cutaneous Adverse Drug Reactions: Are They Still Research Tools or Diagnostic Tests Already? Int J Mol Sci. 2017;18(8).

4. Reantragoon R, Corbett AJ, Sakala IG, Gherardin NA, Furness JB, Chen Z, et al. Antigen-loaded MR1 tetramers define T cell receptor heterogeneity in mucosal-associated invariant T cells. The Journal of experimental medicine. 2013;210(11):2305-20.

5. Corbett AJ, Eckle SB, Birkinshaw RW, Liu L, Patel O, Mahony J, et al. T-cell activation by transitory neo-antigens derived from distinct microbial pathways. Nature. 2014;509(7500):361-5.

Supplementary Figure 1. Flow cytometry gating strategy**. A.** Gating strategy used for flow cytometry analysis of PBMC **(A)** and BFC **(B)**. Lymphocytes were gated by size and granularity, followed by doublet and cell death (7-AAD^-^ ) exclusion allowing identification of IFN-γ+ or CD3+ (T) cells (i) amongst total lymphocytes. A CD19^-^ CD14^-^ gate was applied to exclude monocytes and B cells (ii), and CD3+ cells (red gate) were assessed for γδ TCR expression, binding to MR1 5-OP RU tetramer (MR1 tet) (i.e. MAIT cells), CD45RO expression (i.e. memory), CD69 expression (i.e. activation), CD69 and CD103 co-expression (i.e. tissue-residency/recent egress/memory) and IFN-γ production. Numbers in red indicate the percentage of gated cells (black gate) amongst the parental population except for ii) where the proportion shown is amongst CD3+ cells (defined by the red gate). Plots shown are from Case 3 samples stimulated with PMA and ionomycin (PMA/iono). This gating strategy was also applied to Cases 1 and 4, as shown in Figure 2.

Supplementary Figure 2. Production of IFN-γ by lymphocytes in blood and blister fluid samples**.** BFC and PBMC from Cases 1, 3 and 4 were stimulated with PMA/Iono in the presence of Golgi Plug for 4 hours at 37 °C or cultured in media alone. **A.** 7-AAD^-^ CD14^-^ CD19^-^ lymphocytes were examined for expression of IFN-γ and CD3. **B.** IFN-γ+ lymphocytes from PMA/Ionomycin-stimulated samples (gated as per A) were examined for expression of CD4 and CD8 co-receptors, γδ TCR, binding to MR1 5-OP-RU tetramer (MR1 tet), expression of CD56, which defines NK cells (CD3-CD56+) and is also associated with other unconventional cells NK-like T cells (CD56+CD3+), and expression of CD69 or CD45RO (activation and memory markers respectively). Numbers in red indicate the percentage of the parental population**.** CD45RO plots show IFN-γ+ cells (blue) overlaying total lymphocytes within the same sample (grey).

Supplementary Figure 3. Production of IL-17 by T cells in blood and blister fluid samples**.** BFC and PBMC from Cases 1, 3 and 4 were stimulated with PMA and ionomycin (PMA/Iono) in the presence of Golgi Plug for 4 hours at 37 °C or cultured in media alone. **A.** 7-AAD^-^ CD14^-^ CD19^-^ lymphocytes were examined for expression of IL-17 and CD3.

Supplementary Figure 4. Cytotoxicity assays for Sulfamethoxazole (SMX), Trimethoprim (TMP) and Bactrim. Cryogenically stored healthy donor PBMC were analysed by flow cytometry for % cell death (7-AAD+) using the same experimental conditions as those for ELIspot assays in Figure 1 (after 20h of drug exposure at 37 ^o^C).
