# Supplementary material for: Blister fluid as a cellular input for ex vivo diagnostics in drug-induced severe cutaneous adverse reactions improves sensitivity and explores immunopathogenesis
Source: J Allergy Clin Immunol Glob. 2021 Nov 30;1(1):16–21. doi: 10.1016/j.jacig.2021.11.001 (PMC10509900; doi:10.1016/j.jacig.2021.11.001)
Supplement: Supplementary file 3 [file mmc1.docx]

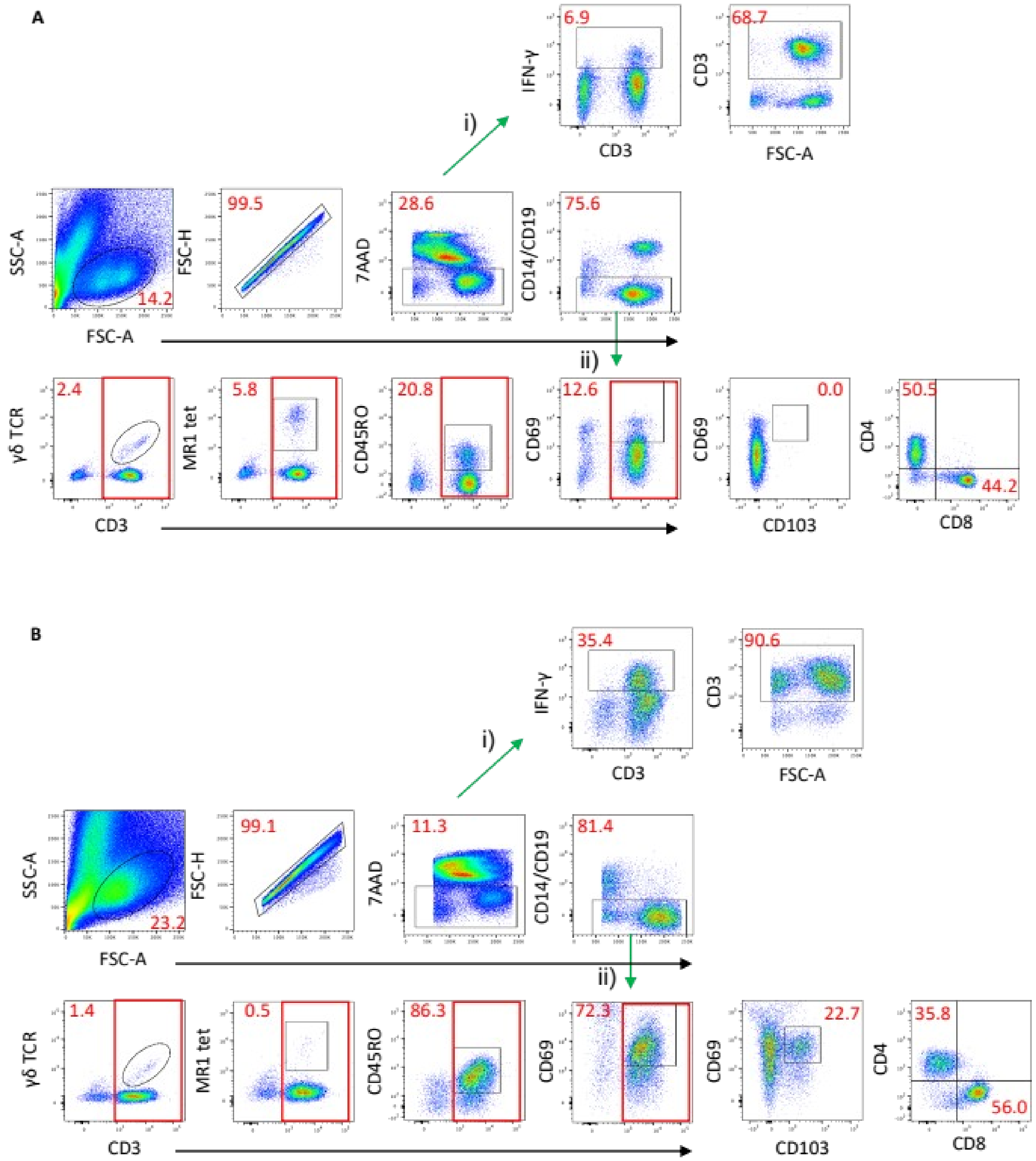
A
i)
ii)
IFN-γ
CD3
6.9
68.7
FSC-A
SSC-A
FSC-H
7AAD
CD14/CD19
14.2
99.5
28.6
75.6
γδ TCR
MR1 tet
CD45RO
CD69
CD4
CD8
CD103
2.4
5.8
20.8
12.6
0.0
50.5
44.2
B
35.4
90.6
23.2
99.1
11.3
81.4
1.4
0.5
86.3
72.3
22.7
35.8
56.0
