# Supplementary material for: Blister fluid as a cellular input for ex vivo diagnostics in drug-induced severe cutaneous adverse reactions improves sensitivity and explores immunopathogenesis
Source: J Allergy Clin Immunol Glob. 2021 Nov 30;1(1):16–21. doi: 10.1016/j.jacig.2021.11.001 (PMC10509900; doi:10.1016/j.jacig.2021.11.001)
Supplement: Supplementary file 4 [file mmc2.docx]

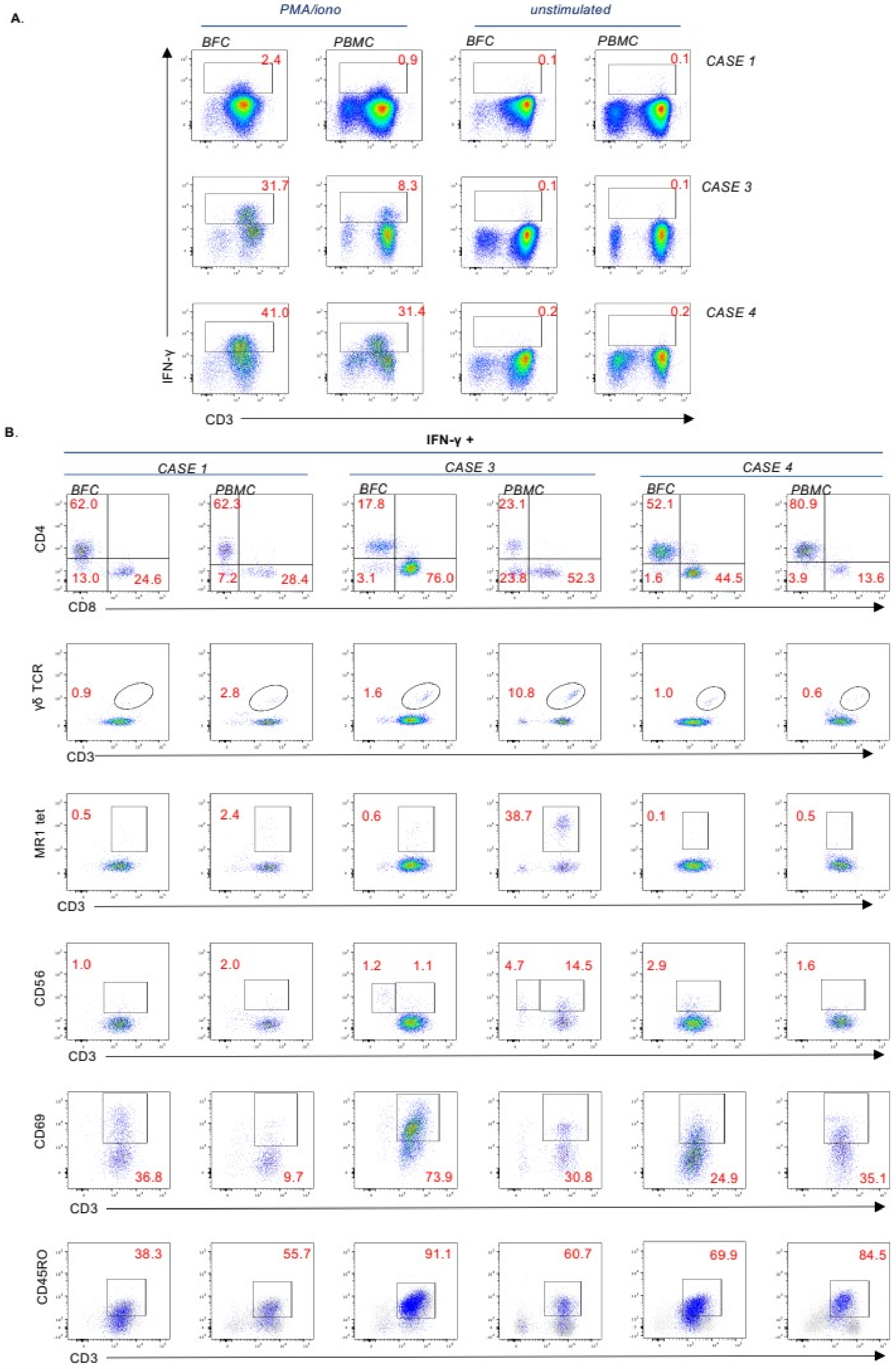

A.
PMA/iono
unstimulated
BFC
PBMC
BFC
PBMC
CASE 1
CASE 3
CASE 4
IFN-γ
CD3
B.
IFN-γ +
CASE 1
CASE 3
CASE 4
CD4
CD8
γδ TCR
CD3
MR1 tet
CD56
CD69
CD45RO
