# Supplementary material for: Blister fluid as a cellular input for ex vivo diagnostics in drug-induced severe cutaneous adverse reactions improves sensitivity and explores immunopathogenesis
Source: J Allergy Clin Immunol Glob. 2021 Nov 30;1(1):16–21. doi: 10.1016/j.jacig.2021.11.001 (PMC10509900; doi:10.1016/j.jacig.2021.11.001)
Supplement: Supplementary file 5 [file mmc3.docx]

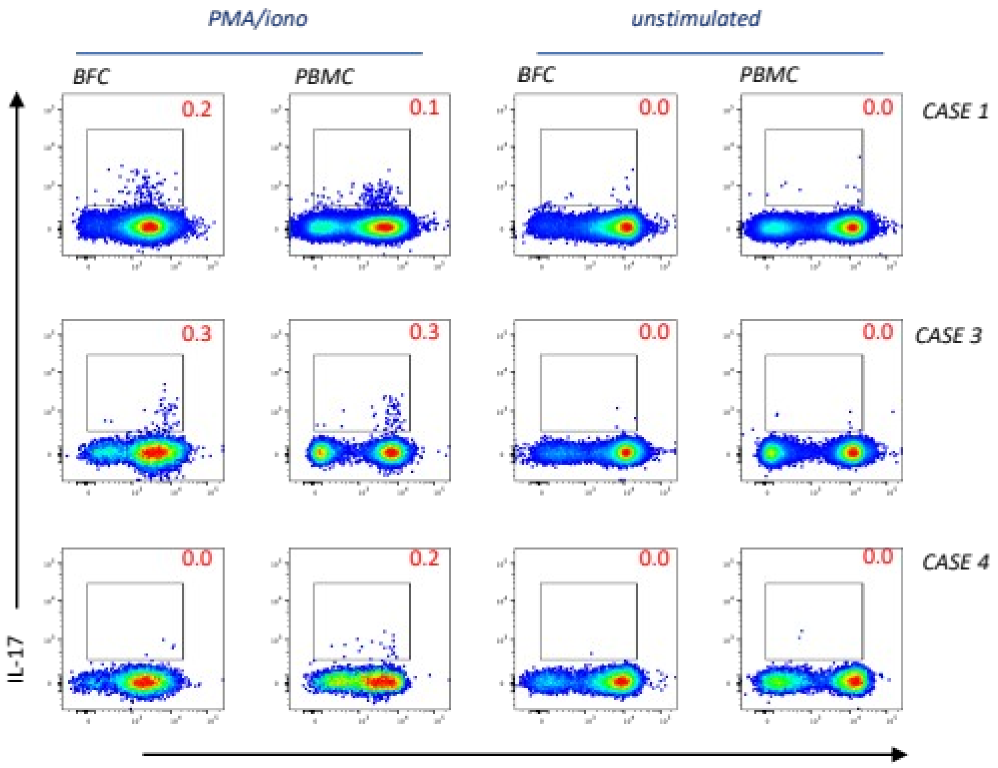
PMA/iono
unstimulated
BFC
PBMC
BFC
PBMC
CASE 1
CASE 3
CASE 4
0.2
0.1
0.0
0.0
0.3
0.3
0.0
0.0
0.0
0.2
0.0
0.0
IL-17
